# Non-coding RNA and n6-methyladenosine modification play crucial roles in neuropathic pain

**DOI:** 10.3389/fnmol.2022.1002018

**Published:** 2022-11-16

**Authors:** Kexin Zhang, Pei Li, Yuanyuan Jia, Ming Liu, Jingjing Jiang

**Affiliations:** Department of Anesthesiology, Shengjing Hospital of China Medical University, Shenyang, China

**Keywords:** n6-methyladenosine (m6A), non-coding RNAs (ncRNAs), neuropathic pain, glial cell, neuroinflammation

## Abstract

After peripheral nerve injury, pain signals are transmitted from primary sensory neurons in the dorsal root ganglion (DRG) to the central nervous system. Epigenetic modification affects neuropathic pain through alterations in the gene expression in pain-related areas and glial cell activation. Recent studies have shown that non-coding RNA and n6-methyladenosine (m6A) methylation modification play pivotal regulatory roles in the occurrence and maintenance of neuropathic pain. Dysregulation of the RNA m6A level *via* dynamic changes in methyltransferase and demethylase after central or peripheral nerve injury commonly regulates pain-associated genes, contributing to the induction and maintenance of neuropathic pain. The dynamic process has significant implications for the development and maintenance of neuropathic pain. However, the underlying mechanisms by which non-coding RNA and m6A RNA modification regulate neuropathic pain are not well-characterized. This article elucidates the multiple mechanisms of non-coding RNA and m6A methylation in the context of neuropathic pain, and summarizes its potential functions as well as recent advances.

## Introduction

Neuropathic pain is a pain disorder caused by a lesion or disease of the somatosensory nervous system, which can be composed of peripheral neuropathic pain and central neuropathic pain (Scholz et al., 2019). The main symptoms of neuropathic pain are allodynia (a painful response to a normally non-painful stimulus) and hyperalgesia (an increased pain from a stimulus that normally provokes pain), which substantially reduce the quality of life of patients, such as through the development of sleep disorders, anxiety, and depression (Baron, 2009). Clinical management strategy for neuropathic pain remains one of the major challenges because painful symptoms are heterogenous and multidimensional (Bouhassira, 2019). Commonly used treatments for neuropathic pain such as antidepressants, anticonvulsants, and analgesics have moderate efficacy and increase the risk of central nervous system side effects. Investigating the molecular mechanisms of neuropathic pain and identifying its specific treatments will be the main directions for future research.

Following peripheral nerve injury, the pain signaling is subsequently transmitted to the central nervous system including the dorsal horn of the spinal cord and pain-related brain regions connected to the nociceptors at the end of the primary sensory neurons in the DRG, resulting in central sensitization (Carlton et al., 2009). There are diverse reciprocal interactions between sensory neurons and glial cells which form a unit linking glial cell activation and spinal sensitization in the context of neuropathic pain. Glial cell activation is linked to neuropathic pain induction and maintenance, playing a crucial role in the transmission of pain signals (Yu et al., 2020; Donnelly et al., 2020). Epigenetic regulation at the gene level has increasingly attracted attention with the expansion of research on neuropathic pain. Epigenetic modifications such as non-coding RNAs (ncRNAs), histone covalent modifications, DNA methylation, and RNA methylation by n6-methyladenosine (m6A) regulate the expression of genes related to axon growth and glial cell activation after peripheral nerve injury (Zhang L. et al., 2021; Liu et al., 2022). Recent studies have unraveled the role of ncRNAs as potential regulators between nerve injury, inflammation, and neuropathic pain (Sommer et al., 2018).

n6-methyladenosine modification is the most prevalent internal modification of eukaryotic mRNAs and occurs primarily with adenine in mRNAs (Meyer et al., 2012). Mediated by methyltransferase, demethylase and m6A-binding protein, m6A regulates mammalian cell proliferation and differentiation *via* mRNA splicing, translation, and stability (Mauer et al., 2017; Zaccara et al., 2019). m6A methylation of mRNA is a gene level modification similar to DNA methylation, that contributes to heritable alterations in gene expression without changing the gene sequence (Zhao et al., 2020). m6A modification exists not only in mRNAs, but also in almost all types of ncRNAs (Zhao et al., 2021). However, the differing roles of m6A on ncRNAs in mammalian cells are less clear. Herein, we aim to investigate the epigenetic mechanism by which ncRNAs and m6A methylation influence nerve injury-induced neuropathic pain.

## Non-coding RNAs and neuropathic pain

### Classification and features of non-coding RNAs

Non-coding RNAs are small RNA molecules that typically do not encode detectable protein. Compared with coding RNAs, ncRNAs functions are more complex. Nearly 70% of the genome is translated to ncRNAs. There are many types of ncRNAs. In this review, we focus on three types of ncRNAs: miRNA (microRNA), lncRNA (long non-coding RNA), and circRNA (circular RNA).

The processing of primary miRNAs with hairpin-like structure into mature miRNAs entails splicing by RNase III proteins, Drosha and Dicer (Lee et al., 2003). The length of endogenous lncRNAs exceeds 200 nucleotides, lncRNAs may bind target genes more specifically and selectively due to their length. LncRNAs can also be categorized into five types with respect to their complimentary protein-coding gene, which can be orientated in a sense and/or antisense direction: (1) sense, (2) antisense, (3) bidirectional, (4) intronic and (5) intergenic (Qi and Du, 2013). CircRNA is the circular non-coding RNA formed by covalent binding of 3′ arm and 5′ arm after reverse splicing. Several studies have demonstrated the involvement of lncRNAs and circRNAs in the regulatory mechanisms involved in the development of neuropathic pain. lncRNAs and circRNAs which have multiple complementary binding sites of miRNAs participate in the development of neuropathic pain principally through acting as competing endogenous RNAs (ceRNAs). ceRNAs sponge their target miRNAs to decrease the expression of target gene of miRNAs (Salmena et al., 2011).

### Non-coding RNAs regulate the expression of pain-associated genes in neuropathic pain

Dysregulation of pain-associated genes is related to epigenetic modification of injured neurons. Moreover, investigations of pain-associated genes by ncRNAs provide insights into the mechanisms underlying neuropathic pain (Table 1).

**TABLE 1:** Non-coding RNAs in different pain models regulate pain-related genes.

Pain model	Dysregulated miRNAs	Regulation	Target	Site	References
pSNL	miR-23a	Downregulation	TXNIP/NLRP3 axis	Spinal glial cell	Pan et al., 2018
CCI	miR-26a-5p	Downregulation	MAPK6	Spinal cord	Zhang et al., 2018
CCI	miR-146a-5p	Upregulation	IRAK1/TRAF6 signaling	DRG, SDH	Wang Z. et al., 2018
CCI	miR-34c	Downregulation	NLRP3	Spinal cord	Xu et al., 2019
SNI	miR-30c-5p	Upregulation	BAMBI	Spinal cord, DRG, CSF, plasma	Tramullas et al., 2018
CCI	miR-381	Downregulation	HMGB1/CXCR4	Spinal cord	Zhan et al., 2018
SNL	miR-124-3p	Downregulation	Egr1	DRG, SDH	Jiang et al., 2021
CCI	miR-34c-5p	Upregulation	SIRT1/STAT3	DRG	Mo et al., 2020
CCI	LncRNA XIST	Upregulation	miR-150/ZEB1 axis	Spinal cord	Yan et al., 2018
CCI	LncRNA XIST	Upregulation	miR-137/TNFAIP1axis	Spinal cord	Zhao et al., 2018
CCI	LncRNA XIST	Upregulation	miR-544/STAT3 axis	Spinal cord	Jin et al., 2018
CCI	LncRNA NEAT1	Upregulation	miR-381/HMGB1 axis	Spinal cord	Xia et al., 2018
CCI	LncRNA CRNDE	Upregulation	miR-136/IL6R axis	Spinal cord	Zhang D. et al., 2019
SNL	LncRNA SNHG1	Upregulation	CDK4	Spinal cord	Zhang J. Y. et al., 2020
SNL	Kcna2 antisense RNA	Upregulation	Zinc finger protein 1/Kcna2	DRG	Zhao et al., 2013
SNL	circAnks1a	Upregulation	miR-324-3p/VEGFB	SDH	Zhang S. B. et al., 2019
CCI	Circ_0005075	Upregulation	miR-151a-3p/NOTCH2 axis	Spinal cord	Zhang Y. et al., 2021
CCI	circRNA zRANB1	Upregulation	miR-24-3p/LPAR3	Spinal cord	Wei et al., 2020

miRNAs are estimated to regulate more than 30% of genes *in vivo* and participate in the development and maintenance of neuropathic pain. Some miRNAs affect neuropathic pain by modulating gene expression and inducing activation of glial cells. Overexpression of miR-34c was shown to reduce the secretion of NLRP3, ASC, caspase-1, IL-1β, and IL-18 in the spinal cord. miR-34c greatly ameliorates neuropathic pain by inhibiting NLRP3-mediated neuroinflammation and cell apoptosis (Xu et al., 2019). Downregulation of miR-34c-5p was found to relieve neuropathic pain through SIRT1/STAT3 (signal transducer and activator of transcription 3) signaling pathway (Mo et al., 2020). Ablation of miR-23a in partial sciatic nerve ligation (pSNL) mice was shown to directly inhibit the expression of CXCR4 on microglia and regulate TXNIP/NLRP3 expression leading to neuropathic pain (Pan et al., 2018). miR-26a-5p is significantly decreased in chronic constrictive injury (CCI) model and in turn downregulates mitogen-activated protein kinase (MAPK) 6, which exacerbates neuropathic pain (Zhang et al., 2018). miR-146a-5p in DRG and SDH was shown to inhibit IRAK1 and TRAF6 through TLRs and IL-1 receptor (TIR) pathway, and finally relieve CCI-induced neuropathic pain (Wang Z. et al., 2018). After sciatic nerve injury (SNI), miR-30c-5p was up-regulated in the spinal cord, DRG, cerebrospinal fluid (CSF), and plasma, while miR-30c-5p was causally involved in neuropathic pain. Analysis indicated that the expression of miR-30c-5p in plasma and CSF may help predict neuropathic pain (Tramullas et al., 2018). Aberrant overexpression of mir-26a-5p suppresses neuropathic pain and neuroinflammation *via* target gene MAPK6 (Zhang et al., 2018). miR-381 in CCI rats showed a negative correlation with high mobility group box 1 (HMGB1) and CXCR4 to regulate neuropathic pain (Zhan et al., 2018). SNL-induced downregulation of miR-124-3p in DRG and dorsal horn is related to up-regulation of Egr1, and affects neuropathic pain (Jiang et al., 2021).

There is a large body of evidence suggesting a crucial role of lncRNAs in the pathophysiology of neuropathic pain (Li T. et al., 2019). Following peripheral nerve injury, dysregulated lncRNAs in the injured neurons were shown to modulate neuropathic pain by regulating the expression of pain-related genes (Jia et al., 2020). lncRNA XIST affects neuropathic pain by sponging target miRNAs to influence pain-related genes. XIST affects the expression of TNFAIP1 gene and may further promote neuropathic pain by specifically sponging miR-137 (Zhao et al., 2018). XIST sponges miR-544 to activate STAT3 (Jin et al., 2018), and XIST modulates the expression of ZEB1 through targeting miR-150 (Yan et al., 2018). XIST is a novel pronociceptive lncRNA in the context of neuropathic pain. In addition to the role of lncRNA NEAT1 in cancer, silencing of NEAT1 can also inhibit neuropathic pain and neuroinflammation. NEAT1 targets miR-381 to affect HMGB1, and induces post-CCI neuropathic pain (Xia et al., 2018). Overexpression of CRNDE, which act as a sponge for miR-136, was found to enhance neuropathic pain and neuroinflammation (Zhang D. et al., 2019). lncRNA SNHG1 blocked SNL-induced neuropathic pain because of the CDK4 level which can regulate inflammatory factors (Zhang J. Y. et al., 2020). After SNL, there is an activation of myeloid zinc finger protein 1 which is a transcription factor that can target the Kcna2 antisense RNA promoter region. The upregulation of Kcna2 antisense RNA, a conserved lncRNA, in injured DRG ultimately suppresses neuropathic pain symptoms (Zhao et al., 2013). This study revealed that endogenous Kcna2 antisense RNA may act as a therapeutic target for neuropathic pain.

In addition, an accumulating body of evidence has shed new light on the relationship of circRNA in DRG and spinal dorsal horn with neuropathic pain. Circ_0005075 contributes to CCI-induced neuropathic pain through sponging miR-151a-3p which regulates Notch2 expression (Zhang Y. et al., 2021). In the cytoplasm, circAnks1a serves as a ceRNA binding miR-324-3p to regulate VEGFB expression. Upregulation of VEGFB increases the excitability of dorsal horn neurons and aggravates neuropathic pain (Zhang S. B. et al., 2019). In CCI rats, LPAR3 expression level was significantly altered by circRNA zRANB1 which is an upstream regulator of miR-24-3p (Wei et al., 2020).

### Non-coding RNAs regulate neuropathic pain *via* glial cell activation

Recently, several studies have demonstrated the molecular mechanism by which miRNAs contribute to neuroinflammation, glial cell migration, and apoptosis. After peripheral nerve injury, miRNAs bind to specific transcription factors or 3′-UTR of target mRNA to dampen mRNA translation and degradation; this alters the cell phenotype and the activation state of glial cells. miRNAs such as miR-124-3p (Jiang et al., 2020) and miR-210 (Li B. et al., 2020) can respectively promote and inhibit the activation of glial cells. miR-155 activates microglia by regulating inflammation-related gene SOCS1 and inflammatory cytokines (Tan et al., 2015). miR-124 was found to control the activation of microglia by inhibiting p65- and TRAF6-dependent TLR signaling (Qiu et al., 2015). Intrathecal injection of miR-124 was shown to alleviate M1/M2 type markers of microglia after nerve injury, which is a potential tool for the treatment of neuropathic pain (Willemen et al., 2012).

lncRNAs can act on microglia and astrocytes to trigger neuropathic pain (Liu et al., 2020). Significant upregulation of lncRNA SNHG5 in SNL model promotes the activation of microglia and astrocytes and exacerbates neuropathic pain by targeting miR-154-5p/CXCL13 (Chen M. et al., 2020). In CCI, lncRNA FIRRE was upregulated in the spinal cord. It contributes to neuropathic pain in mice by increasing HMGB1expression in microglia (Wen et al., 2021). LncRNA PVT1 is believed to target miR-186-5p/CXCL13/CXCR5 axis, and is responsible for neuropathic pain caused by overactivation of astrocytes (Zhang P. et al., 2021). Sponging of miR-219-5p by lncRNA XIST inhibited the viability of microglia cells, thereby affecting neuropathic pain (Zhong et al., 2021). In recent studies, antisense lncRNAs were also found to affect neuropathic pain. After total brachial plexus root avulsion (tBPRA), the downregulation of lncRNA JHDM1D antisense 1 (JHDM1D-AS1) may act as a ceRNA negatively regulating miR-101-3p. JHDM1D-AS1 can suppress microglial inflammation to influence neuropathic pain *via* miR-101-3p (Liu et al., 2020).

**FIGURE 1:**
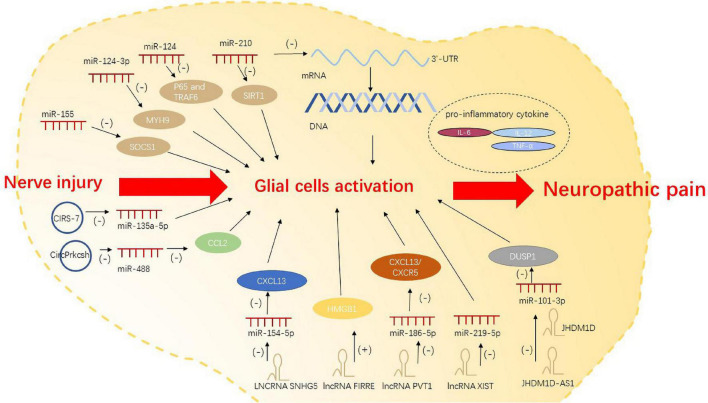
Non-coding RNAs regulate neuropathic pain *via* glial cell activation.

circRNAs can also act as ceRNAs to inhibit miRNA expression, and then regulate the pro-inflammatory genes and the activation of glial cells. Studies have identified a variety of regulatory pathways by which circRNAs regulate the activation and polarization of microglia (Li M. et al., 2021; Jiang et al., 2022). In a study, the expression of CIRS-7 in spinal dorsal horn showed a positive correlation with the occurrence of CCI-induced neuropathic pain. Binding of CIRS-7 with miR-135a-5p was shown to enhance the activation of microglia by promoting the pro-inflammatory cytokines IL-6, IL-12, and TNF-α (Cai et al., 2020). Lastly, spinal cord injury(SCI)-induced inflammatory response was shown to be inhibited by CircPrkcsh through the miR-488/CCL2 axis in astrocytes (Chen et al., 2022; Figure 1).

## n6-methyladenosine methylation and neuropathic pain

### Structure and molecular function of n6-methyladenosine

#### n6-methyladenosine “writers”- methyltransferases

Methyltransferases, also known as m6A “writers” increase the expression of m6A. This family of enzymes consists of METTL3, METTL14, and Wilms’ tumor 1-associating protein (WTAP) (Bokar et al., 1997). Methyltransferases form a methyltransferase complex and function in conjunction with each other. The enzymes can be identified by diverse M6A “readers” through YTH domain proteins, indicating the effects of m6a are dynamic and reversible. The activity of the methyltransferase complex is also affected by miRNAs (Chen et al., 2015).

#### n6-methyladenosine “erasers”- demethylases

Currently, there are two known m6A-specific demethylases, FTO, and ALKBH5. FTO, a fat-mass and obesity-associated protein, participates in RNA m6A modification. FTO belongs to the ALKB dioxygenase family and is dependent on α-Ketoglutarate (α-ketoglutaric acid, α-Kg) and divalent iron ions located in both the nucleus and cytoplasm. m6A can be restored to adenosine under the enzymatic action of the m6A RNA demethylase FTO or ALKBH5 in mammals, indicating that m6A modification is dynamically reversible (Jia et al., 2011). The substantial increase of FTO identified in injured DRG tissue after peripheral nerve injury suggests that FTO may be a key target of neuropathic pain in future research (Li Y. et al., 2020).

#### n6-methyladenosine “readers”- n6-methyladenosine -binding proteins

n6-methyladenosine “readers” refers to a large class of reading proteins, such as YTH domain family proteins (m6A-binding protein YTH domain family) and insulin like growth factor 2 mRNA binding proteins (IGF2BP family proteins). These readers can recognize methylated mRNAs and directly or indirectly combine with them to promote m6A methylation together with methyltransferase. YTH domain family proteins may affect the pathological process of neuropathic pain by regulating RNA metabolism in terms of transcription, translation, splicing, transportation, and degradation.

### n6-methyladenosine methylation regulates the expression of pain-associated genes in neuropathic pain

The imbalance of methylase and demethylase can modify m6A level, which regulates the transcription of pain-associated genes and the release of inflammatory mediators after nerve injury, finally affecting neuropathic pain.

#### METTL3 regulates the expression of pain-associated genes

n6-methyladenosine deletion caused by cerebellar conditional knockdown of METTL3 reduces the half-lives of the mRNAs of development- and apoptosis-related genes and triggers abnormal splicing of the mRNAs of synapse-associated genes, resulting in abnormal cell differentiation and apoptosis (Wang C. X. et al., 2018). Many studies have indicated the involvement of apoptosis in nerve damage and the process of development of neuropathic pain (Zhou et al., 2019; Chen X. J. et al., 2020).

It is well established that specific suppressors of the spinal cord such as METTL3 and YTHDF2 have a positive correlation with the upregulation of TET1 which is a DNA demethylase (Albik and Tao, 2021). TET1 overexpression relieves neuropathic pain *via* affecting the μ-Opioid receptor (MOR) and Kv1.2 expression to modulate pain-associated genes (Wu et al., 2019). Data available in the literature indicate that the downregulation of METTL3 has a specific positive correlation with the downregulation of m6A modification of TET1 mRNA in the spinal cord, subsequently resulting in the reduction of YTHDF2 binding to TET1 mRNA in the complete Freund adjuvant (CFA) model. CFA reduced both YTHDF2 and its target binding site in TET1 mRNA (Pan et al., 2021b), and the increased expression of TET1 mRNA contributes to neuropathic pain. YTHDF2 reduces the demethylation of the brain-derived neurotrophic factor (BDNF) gene by repressing TET1 expression, and the inhibited expression subsequently reduces pain (Hsieh et al., 2016).

#### FTO regulates the expression of pain-associated genes

**FIGURE 2:**
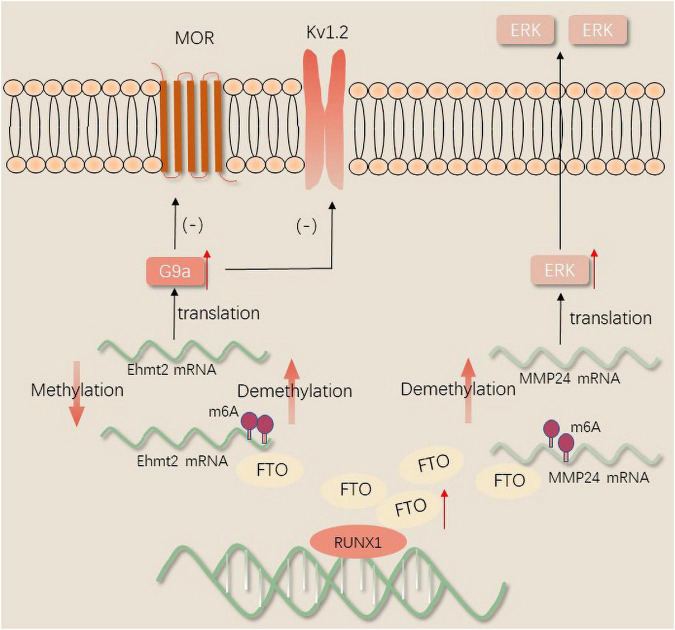
The mechanism of FTO regulates neuropathic pain.

FTO in the injured DRG may contribute to neuropathic pain by stabilizing the differential expression of G9a, a histone methyltransferase encoded by EHMT2 mRNA that can remarkably inhibit transcription (Laumet et al., 2015). Transcription activator Runx1 was reported to increase the expression of FTO in the DRG in a model of SNL. After peripheral nerve injury, Runx1 may be responsible for the loss of the m6A site in the EHMT2 mRNA encoding G9a through binding to FTO gene promoter and increasing the FTO gene expression in the injured DRG. The upregulation of the G9a protein in the DRG induces and maintains neuropathic pain by reducing the amount of MOR in the injured DRG (Li Y. et al., 2020). A recent study revealed that the gene silencing of potassium channels and the downregulation of Kv1.2 promote peripheral nerve injury-induced neuropathic pain through selective knockdown of G9a in the DRG (Zheng et al., 2021). Nerve injury results in a loss of MOR expression in DRG and spinal cord neurons. Therefore, opioids exhibit a lower analgesic effect on neuropathic pain. Recently, it was found that FTO was correlated with MMP24 expression in spinal cord neurons. FTO binding to MMP24 mRNA following SNL diminishes the m6A enrichment in MMP24 mRNA, subsequently accelerates the translation of MMP24 to activate extracellular signal-regulated protein kinases(ERKs), and ultimately resulting in neuropathic pain (Ma et al., 2021; Figure 2).

### n6-methyladenosine regulates neuropathic pain *via* glial cell activation

Glial cells mainly consist of oligodendrocytes, astrocytes and microglia. The activation mechanism of glial cells provides some insights into the mechanism of neuropathic pain (Navia-Pelaez et al., 2021). Microglia activation-induced neuroinflammation is a major component of neuropathic pain. The upregulation and activation of the P2 × 4 receptor and protein kinase p38 MAPKs in microglia can provoke neuropathic pain after peripheral nerve injury (Williams et al., 2019; Mai et al., 2020). This provides evidence that injured primary afferent neurons release neurotransmitters and activate microglia; for example, colony stimulating factor 1 (CSF1) from the injured DRG binds to the CSF1 receptor (CSF1R) on the microglia to activate and proliferate (Guan et al., 2016). Conditional deletion of cholesterol transporters ABCA1 and ABCG1 in microglia occurs in response to neuroinflammation and neuropathic pain (Navia-Pelaez et al., 2021). Overexpression of the m6A methyltransferase METTL3 increases the levels of the pro-inflammatory cytokines, and promotes the lipopolysaccharide (LPS) induced microglial inflammatory response by activating the TRAF6-NF-κB pathway (Wen et al., 2022). Conditional knockdown of YTHDC1 increases the markers and mediators of pro-inflammatory phenotype (M1) microglia by elevating the phosphorylation of STAT3 (Zhou et al., 2021). The rapid aggregation and activation of spinal glial cells that trigger neuroinflammation suggest that the bidirectional interactions of spinal glial cells affect the development of neuropathic pain (Yu et al., 2020).

There are differences in the role of astrocytes and microglia in neuropathic pain (Chen et al., 2018) as well as differences in their activation processes and duration. Microglia are activated immediately after the nervous system injured. Microglia activation precedes astrocyte activation, and astrocytes play a persistent role thereafter (Ji et al., 2013). Evidence suggests that astrocyte-mediated neuroinflammation shows efficacy in the maintenance of neuropathic pain. Astrocytes proliferate and show morphological and functional changes after nerve injury. Astrocytes present several changes such as activation, releasing inflammatory mediators and leading to neuroinflammation and neuropathic pain (Li Z. et al., 2019). The expression of METTL3 in astrocytes was increased in mouse SCI model (Xing et al., 2021). IL-33 levels in oligodendrocytes in the dorsal spinal cord were found to be elevated after SCI, and intrathecal injection of IL-33 enhanced pain hypersensitivity after painful injury through TNF-α and IL-1β (Zarpelon et al., 2016). The role of oligodendrocytes in neuropathic pain regulation is mediated by interaction with microglia and astrocytes. Glial cells can also form a positive feedback loop through intercellular interaction to develop and maintain neuropathic pain. The mediators secreted by glial cells, such as cytokines and chemokines, lead to mechanical and thermal hyperalgesia. For instance, the chemokine CX3CL1 regulates neuropathic pain through neuron-microglia interactions, CCL2 and CXCL1 regulates neuropathic pain through astrocyte-neuron interactions in the spinal cord, and CXCL13 promotes neuropathic pain through neuron-astrocyte interactions (Zhang et al., 2017).

## n6-methyladenosine regulates neuropathic pain *via* non-coding RNAs

m6A methylation not only regulates target mRNAs, but also ncRNAs to participate in diverse biological processes. The crosstalk of m6A and ncRNAs is pervasive and inspiring, extending the scope of epigenetics. m6A methylation has been shown to regulate miRNA maturation and further affect neuropathic pain in *in vitro* (Alarcón et al., 2015). METTL3 is sufficient to methylate massive pri-miRNAs to reinforce miRNA maturation. In spared nerve injury state, ablation of METTL3 was associated with m6A on primiR-150. The underlying mechanism involved cooperation of METTL3 with YTHDF2 accelerating miR-150 maturation. The maturation of primiR-150 was inhibited by DGCR8, and primiR-150 further impaired the target protein BDNF to promote neuropathic pain (Zhang et al., 2022). DGCR8 which is a microprocessor complex subunit initiates miRNA synthesis and maturation (Quick-Cleveland et al., 2014). Silencing of METTL3 can inhibit the maturation of miR-7212-5p (Mi et al., 2020). In the CFA-induced neuropathic pain condition, overexpression of METTL3 in the spinal cord, which increases the levels of m6A, influences nociceptive sensitization by primiR-365-3p, which is recognized by DGCR8 (Zhang C. et al., 2020). Additionally, METTL14-mediated m6A modification can regulate the maturation of primiR-126 (Ma et al., 2017).

**FIGURE 3:**
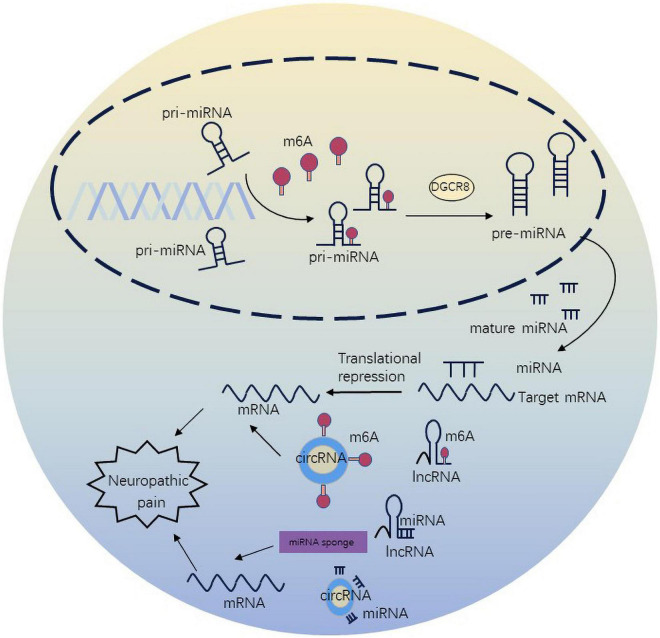
n6-methyladenosine methylation regulate non-coding RNAs to affect neuropathic pain.

n6-methyladenosine modulates the expression and stability of lncRNAs. METTL3-mediated m6A modification increased the expression level of lncRNA MALAT1. At the same time, METTL3/YTHDF3 complex increased the stability of lncRNA MALAT1 (Jin et al., 2019). MALAT1 can also disturb the development and occurrence of neuropathic pain through the miR-206/ZEB2 axis (Chen et al., 2019). YTHDC1 has been illustrated to preferentially bind m6A sites in diverse ncRNAs, such as XIST, NEAT1 and MALAT1, but its function has only been probed in the case of XIST (Patil et al., 2016). m6A-driven translation is highly abundant in circRNAs. Mechanistically, m6A initiates the protein synthesis of circRNA by recruiting YTHDF3 and initiation factors eIF4G2 (Yang et al., 2017). circ_0008542 can bind to miR-185-5p by its own m6A methylation modification mediated by ALKBH5 or METTL3 (Wang W. et al., 2021). miR-185-5p attenuates CCI-induced neuropathic pain and neuroinflammation by co-targeting MyD88 and CXCR4 (Huang et al., 2022). METTL3 was found to stabilize circux1 through m6A methylation modification. Circux1 sponges caspase1 mRNA and inhibits caspase1-mediated neuroinflammation and neuropathic pain (Wu et al., 2021; Figure 3).

## Discussion

Neuropathic pain can adversely impair the quality of life of patients. Exploration of the complex pathogenetic mechanism of neuropathic pain is inherently challenging. The transcriptional and translational changes in DRG, spinal dorsal horn, and other pain-related areas (including cortex and dorsal hippocampus) after peripheral nerve injury are believed to be involved in the causation of neuropathic pain (Wang N. et al., 2021; Wei et al., 2021). An increasing number of studies have suggested that ncRNAs can affect neuropathic pain by regulating transcription factors after peripheral nerve injury. miRNAs regulate gene expression by directly binding to the 3’-UTR of the target mRNA, resulting in translational repression or degradation of the mRNA. miR-122-5p directly inhibits PDK4 expression suppressing the progression of neuropathic pain (Wan et al., 2021). In addition, miRNAs regulate transcription *via* regulating the binding of DNA and protein. Loss of miR-30a-3p was found to modify the level of BDNF and acetylated histone H3 and H4 in CCI rats. Moreover, miR-30a-3p was shown to target E-cadherin transcriptional activator (EP300), which modulated BDNF through enhancing acetylated histone H3 and H4 on the promoter (Tan et al., 2020). Histone methylation-mediated miR-32-5p down-regulation regulated trigeminal neuropathic pain by targeting Cav3.2 channels (Qi et al., 2022). LncRNAs, as regulators, modulate the interactions between transcription factors and target genes. Downregulation of DRG-specifically enriched lncRNA (DS-lncRNA) promotes Ehmt2 transcription in injured DRG and likely contributes to neuropathic pain. This indicated that DS-lncRNA downregulation increases the binding of RALY to RNA polymerase II and the Ehmt2 gene promoter (Pan et al., 2021a). Both lncRNAs and circRNAs can regulate glial activation and expression of the pro-inflammatory genes by sponging pain-related miRNAs. As a ceRNA, it induces glial cells to release inflammatory factors, to further regulate neuropathic pain. Besides as miRNA sponges, lncRNAs and circRNAs can interact with many different RNA-binding proteins (RBP) to play several roles in biological processes such as neuropathic pain (Zhang Z. et al., 2021).

In addition to DNA methylation and histone modification, studies have also highlighted the role of m6A methylation in neuropathic pain. m6A modification was shown to regulate the activation of glial cells at the epigenetic level. KEGG analysis showed that the main pathways of mRNA methylation in the activated microglia were the pathways regulating the immune system processes (RAS, HIF-1, TNF, mTOR, Notch, ErbB, FoxO, JAK-STAT, and MAPK signals) and signal transduction pathways (chemokines, RAS, NOD/TLR, B cell/T cell receptors, IL-17) (Li Q. et al., 2021). These data can help inform further studies of the detailed mechanisms by which m6A methylation affects neuropathic pain through glial cell activation. The recognition of the role of m6A methylation modification has changed the traditional research ideas and has become one of the contemporary research hotspots.

There is a bidirectional network between m6A methylation and ncRNAs. m6A level affects the gene expression by affecting the m6A methylation of ncRNAs. For example, ablation of METTL3 was found to negatively affect the maturation of primiR-7212-5p, and then alter the expression of miR-7212-5p (Mi et al., 2020). METTL3-mediated m6A induces upregulation of lncRNA LINC00958 by binding to miR-3619-5p (Zuo et al., 2020). Moreover, ncRNAs can also regulate m6A methylation level. miRNA can regulate the binding of METTL3 to the target site of miRNA (Chen et al., 2015). We surprisingly found that overexpression of miR-124a and miR-155 results in neuropathic pain *via* repressing the expression of target gene SIRT1 to promote differentiation of T cells toward anti-inflammatory phenotype (Heyn et al., 2016). Conversely, YTHDC1 deficiency decreased the stability of SIRT1 mRNA, resulting in microglia polarization and inflammatory response (Zhou et al., 2021). ncRNAs and m6A methylation modification act on the same target gene, but cause different effects. The underlying mechanisms of this phenomenon also need further exploration.

Recent years have witnessed rapid advances in high-throughput RNA sequencing (RNA-seq) and bioinformatics algorithms. Recent studies have shown decreased expression of miR-183 in CCI rat models, which was involved in the progression of neuropathic pain. In addition, increased miR-183 expression was shown to negatively regulates its downstream target MAP3K4, which leads to the downregulation of inflammatory cytokines correlated with neuropathic pain. This suggests that miR-183 is a potential biomarker and therapeutic target for neuropathic pain (Huang and Wang, 2020). Linc00311 and lncRNA-AK141205 were upregulated in bCCI rats. *In vitro* and *in vivo* experiments also indicated that they activated the STAT3 signaling in spinal microglia. Silencing of linc00311 and lncRNA-AK141205 may be a promising treatment for neuropathic pain (Pang et al., 2020). Significant downregulation of METTL3 and m6A methylation was found both in spared nerve injury-induced neuropathic pain rats and Shingles-induced neuropathic pain patients. Serum METTL3 could may serve as an unfavorable prognostic marker in patients with neuropathic pain. It may also be helpful for dynamic monitoring of diseases after treatment of neuropathic pain (Zhang et al., 2022). Detection of dysregulated m6A levels may provide a promising method for the diagnosis, prediction, and assessment of neuropathic pain in future. Overall, more in-depth understanding of ncRNAs and m6A modification biogenesis, and their effects might aid the potential application of diagnostic biomarkers.

Overcoming drug resistance through regulation of pain-related genes may provide an alternative strategy. Achieving *in vivo* delivery of ncRNAs, like other biomolecules, is a major challenge. Currently, the most direct way to inhibit miRNAs is to use antisense oligonucleotides, also named miRNA blockers, that are complementary to the mature miRNAs. However, the exogenous RNA fragment is unstable before entering the cell and is highly susceptible to degradation by nucleases. The use of viruses (e.g., retroviruses, lentiviruses, adenoviruses, adeno-associated viruses) as gene vectors is an efficient method for transfection, but also has many problems, such as oncogenicity, immunogenicity, poor cellular targeting, and localization, limited gene loading capacity, difficulty in vector preparation, and environmental risks. Nowadays, the most commonly researched non-viral vectors are polymers, lipids, peptides, inorganic particles, and hybrid systems (Zu and Gao, 2021). However, non-viral carriers have limitations with respect to the ability to penetrate nuclear membranes, transfection efficiency, and protein expression after transfection. Therefore, development of specific, stable, efficient and safe non-viral vectors is a key imperative. Recently, Pfizer-BioNTech used the BNT162b2 COVID-19 vaccine successfully using mRNA-based lipid nanoparticles which showed 95% effectiveness in preventing COVID-19 (Polack et al., 2020). With the development of material science, especially nanobiotechnology, the delivery of nanoparticles containing tissue-specific miRNA antagomir can target neuron-glial cell communication.

The epigenetic mechanism of ncRNAs and m6A modifications in neuropathic pain needs to be further investigated. This would help to achieve the goal of personalized medical treatment, and provide a theoretical basis for the optimization of clinical therapy and the exploration of new therapies.

## Author contributions

KZ made the substantial contributions to the manuscript text. PL and YJ prepared the Figures 1–3. JJ and ML provided the editing and writing assistant. All authors reviewed the manuscript and approved the submitted version.
